# H1N1 Influenza Patient Saved by Extracorporeal Membrane Oxygenation: First Report from Iran 

**Published:** 2016-07-06

**Authors:** Alireza Jahangirifard, Zargham Hossein Ahmadi, Majid Golestani Eraghi, Payam Tabarsi, Majid Marjani, Afshin Moniri, Seyed Ali Reza Nadji, Seyed Mohammad Reza Hashemian, Ali Akbar Velayati

**Affiliations:** *National Research Institute of Tuberculosis and Lung Diseases (NRITLD), Shahid Beheshti University of Medical Sciences, Tehran, Iran.*

**Keywords:** *Influenza A virus, H1N1 subtype*, *Respiratory insufficiency*, *Extracorporeal membrane oxygenation*

## Abstract

Respiratory failure is a serious complication of H1N1 influenza that, if not properly managed, can cause death. When mechanical ventilation is not effective, the only way to save the patient’s life is extracorporeal membrane oxygenation (ECMO). A prolonged type of cardiopulmonary bypass, ECMO is a high-cost management modality compared to other conventional types and its maintenance requires skilled personnel. Such staff usually comprises the members of open-heart surgical teams. Herein, we describe a patient with H1N1 influenza and severe respiratory failure not improved by mechanical ventilation who was admitted to Masih Daneshvari Medical Center in March 2015. She was placed on ECMO, from which she was successfully weaned 9 days later. The patient was discharged from the hospital after 52 days. Follow-up till 11 months after discharge revealed completely active life with no problem. There should be a close collaboration among infectious disease specialists, cardiac anesthetists, cardiac surgeons, and intensivists for the correct timing of ECMO placement, subsequent weaning, and care of the patient. This team work was the key to our success story. This is the first patient to survive H1N1 with the use of ECMO in Iran.

## Introduction

A new strain of influenza A virus (H1N1) (of swine origin) was identified in 2009 and became pandemic after a few months.^1^ In most patients, the infection presents with mild, self-limiting upper respiratory tract infection. However, in 20% of patients, it may rapidly progress to life-threatening respiratory failure and cause refractory hypoxemia and hypercapnia.^2^ Mechanical ventilation may be indicated in 80% of these patients,^3^ and the administration of extracorporeal membrane oxygenation (ECMO) may be necessary to prevent death.^4^

## Case Report

A 35-year-old previously healthy lady developed sudden-onset rhinorrhea, accompanied by fever, dyspnea, and myalgia. She was admitted to another hospital, where she received intravenous ceftriaxone (1 g) every 8 hours. Nevertheless, progressive exacerbation of the patient’s condition led to her transfer to the emergency room of Masih Daneshvari Medical Center (National Research Institute of Tuberculosis and Lung Disease) in March, 2015.

On examination, the patient was conscious and oriented to time, place, and person. The vital signs are shown in Table 1. Auscultation of the chest revealed bilateral crackles and rhonchi in both lung fields. There were no other positive findings on physical examination.

**Figure 1:**
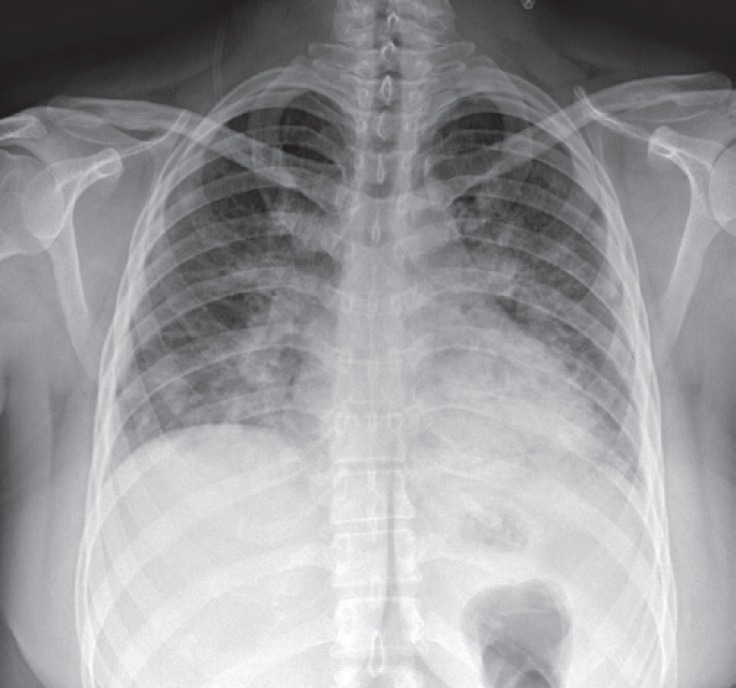
Chest X-ray (posteroanterior view) shows ill-defined patches of alveolar opacities, more in central lung fields and the para-cardiac region. Blunting of the costophrenic angles is evident from the small amount of pleural effusion.

An oxygen mask with 5 liters of oxygen/min was administered, and the saturation was increased to 75%. Chest radiography showed bilateral patchy infiltration (Figure 1). The laboratory data of the patient are illustrated in Table 2.

**Table 1:** Vital signs of the patient at the time of admission

Pulse rate	107/min
Respiratory rate	30/min
Blood pressure	130/80 mmHg
Temperature (orally)	39 ^°^C
Oxygen saturation	65%

**Table 2:** Lab data of the H1N1 patient at the time of admission

Hemoglobin	11.8 g/dL	
White blood cells	2500/mm^3^	
	Polymorphonucleide	60%
	Lymphocyte	35%
Platelets	138000/mm^3^	
Creatinine	0.8 g/dL	

**Figure 2:**
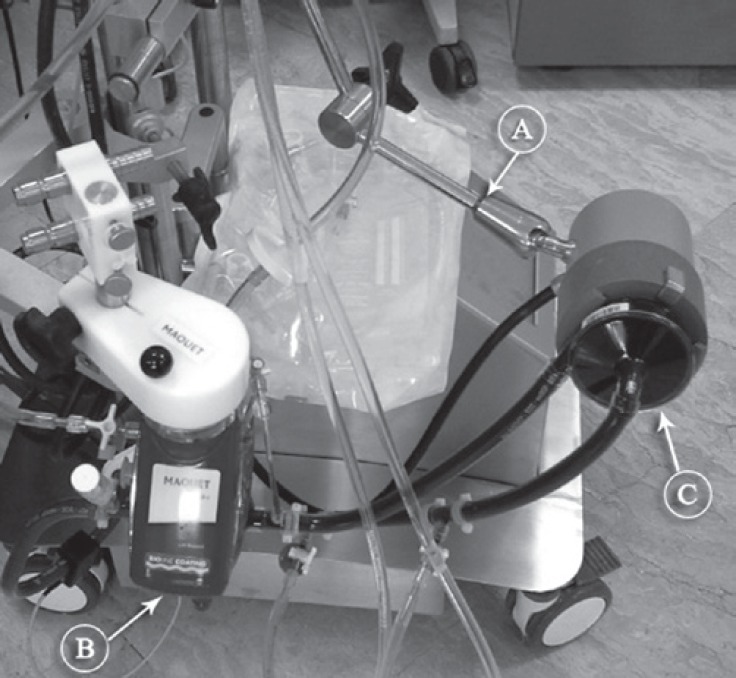
Stockert® SCPC console of ECMO (a) has been assembled with a Maquet PLS oxygenator (b) and its centrifugal pump has been replaced with a Sorin revolution pump (c).

The H1N1 influenza virus was confirmed by the reverse-transcriptase polymerase chain reaction assay of the patient’s respiratory secretions.

The patient was intubated, and mechanical ventilation was instituted. Oseltamivir (150 mg) via a nasogastric tube every 12 hours and broad-spectrum antibiotics-including intravenous ciprofloxacin (400 mg) every 12 hours, intravenous meropenem (1 g) every 8 hours, and vancomycin (1 g) every 12 hours-were started. Due to impaired gas exchange, inability to increase PaO_2 _within 24 hours, and progressive deterioration, she was referred for ECMO implantation. 

An ECMO console (Stockert^®^ SCPC, Sorin Group USA, Inc., 14401 W, 65th Way, Arvada CO 80004, USA) was transported to the intensive care unit of the infectious disease ward, and VV ECMO was applied via the cannulation of the right femoral vein and the right internal jugular vein. Cannulation of the femoral vein and the right internal jugular vein was done with 17-F and 19-F Bio-Medicus cannulae (Medtronic Inc., Minneapolis, MN, USA), respectively. At the time of study, Sorin oxygenators were not available in Iran and the only available oxygenators were QuadroxD (Maquet Cardiovascular, Wayne, NJ, USA) polymethylpentene oxygenators. Since the head pumps of these 2 systems were different, a Sorin Revolution centrifugal pump (Sorin Group Italia, Via Statale 12 Nord, 8641037 Mirandola [MO], Italy) was replaced and assembled to the Maquet oxygenator (Figure 2). After the institution of ECMO, the ventilator settings were reduced to “rest” levels (ventilator frequency = 8 - 10 breaths per minute, peak inspiratory pressure = 25 - 30 cm H_2_O, I/E = 1/1, and fractional inspired oxygen = 0.3 - 0.5) while maintaining positive end-expiratory pressure and 10-15 cm H_2_O to promote lung aeration. Epinephrine (0.05 mic/kg/min) was started after the initiation of ECMO and tapered off after 6 hours. The flow of ECMO was kept between 2500 and 3100 cc/min while the mean arterial pressure was 80 mmHg. Activated clotting time was kept between 180 and 200 seconds and checked every 4 hours, while the infusion of heparin was adjusted. Arterial blood gas was taken every 6 hours, arterial oxygen tension was kept at 90 - 110 mmHg, and arterial carbon dioxide tension was maintained between 30 and 40 mmHg. Additionally, hematocrit was maintained at over 35% and platelet over 80 000. Feeding was started via a nasogastric tube with a supplement of 40 Kcal/kg. With the initiation of ECMO support, the mode of mechanical ventilation was set at continuous mandatory ventilation (ventilator frequency of 10/min and fraction of inspired oxygen [FiO_2_] of 40%) with a peak inflation pressure of 30 cm H_2_O. Tracheostomy was done on the 6th postoperative day. Gradually, the flow of ECMO was decreased with no alteration in oxygen and arterial oxygen tension saturation. ECMO weaning was started as from the 8th postoperative day, and complete weaning and explantation of ECMO was performed on the 9th day.

The patient was weaned off the ventilator on the 16th day, but due to muscle wasting and weakness, she was intermittently dependent on the mechanical ventilator. She was discharged 53 days after admission. Nine months’ follow-up revealed complete health with no complication.

## Discussion

H1N1, which is also referred to as the “swine flu”, can have a rapidly progressive course, causing adult respiratory distress syndrome (ARDS).^1^^, ^^2^ This situation necessitates mechanical ventilation for the management of life-threatening hypoxemia; nonetheless, when the lung damage is so severe that mechanical ventilation is not able to provide sufficient oxygenation of blood, the application of EMCO may be helpful.

We requested ECMO 24 hours after commencing mechanical ventilation and establishing the ineffectiveness of this mode. Although ECMO should be started in the absence of response within hours after starting ventilation, ECMO was not used routinely in our center in that period of time. ECMO is an expensive mode of treatment.^5^^, ^^6^ The ECMO program was started in 2009 at our center, but it coincided with the nuclear crisis and subsequent intense limitations in the import of medical devices.^7^^, ^^8^ However, we succeeded in finding different types of ECMO oxygenators that were available at the time and assembling them onto other types of ECMO consoles: this was the only effective way to save patients. 

Recently, the application of ECMO in ARDS has gained wider popularity and it is even deemed one of the therapeutic modalities in H1N1.^9^ The progression of H1N1 in some patients may be so fast that in less than 24 hours, it causes respiratory failure and subsequent death. Thus, the threshold of the decision for ECMO should be very low. The shorter the duration between the intubation of the patient and ECMO, the better the result.^10^ A review of early ELSO H1N1 registry data from October 2009 revealed 72% survival when ECMO was instituted within 6 days of intubation and 31% survival within 7 days or longer. The duration of ECMO depends on the reversibility of lung function, severity of illness, and protocol of the management of the lungs under ECMO. Different studies have reported a wide range-from 8 days in an American group to 23 days in a French group.^11^ The duration of ECMO in our study was 9 days, which was the longest period of ECMO in our center as at April 2015. It has been previously reported that the incidence of increases when the duration of EMCO exceeds 14 days.^10^ If ECMO is considered in a critically ill H1N1 patient and a center lacks the necessary experience and preparation for it, it is advisable that the patient be referred to a well-trained center.^12^

## Conclusion

ECMO is a complex technique and requires thorough provision, appropriate equipment, institutional commitment, practice, and a dedicated team. Low threshold is necessary for applying ECMO in critically ill H1N1 patients. ECMO supports the patient while the lungs are failing, eliminates the risk of detrimental mechanical ventilation, and allows more time for the inflammation to abate and the lungs to recover. ECMO affords one to “buy time” so as to consider diagnosis and treat the pathology when lung or heart failure is not permanent.

At present, ECMO is not a commonly used modality in Iran and many specialists may not be familiar with extracorporeal perfusion. A successful ECMO program requires close collaboration among cardiac anesthesiologists, intensivists, infectious disease specialists, cardiac or vascular surgeons, perfusionists, and open-heart surgery nurses.

## Notes:

***This paper should be cited as:*** Jahangirifard A, Ahmadi ZH, Golestani Eraghi M, Tabarsi P, Marjani M, Moniri A, Nadji SAR, Hashemian SMR, Velayati AA. H1N1 Influenza Patient Saved by Extracorporeal Membrane Oxygenation: First Report from Iran. J Teh Univ Heart Ctr 2016;11(3):153-156.
